# Percutaneous atrial septostomy for left ventricular unloading in patients on peripheral venoarterial extracorporeal membrane oxygenation: A systematic review and meta-analysis

**DOI:** 10.1016/j.ahjo.2025.100542

**Published:** 2025-04-09

**Authors:** Mohammed AlGhamdi, Gabriel Saiydoun, Guillaume Lebreton, Jean-Philippe Mazzucotelli

**Affiliations:** a Cardiac Surgery Division, King Abdulaziz University, Jeddah, Saudi Arabia; b Cardiac Surgery Division, Pitié-Salpêtrière Hospital, Sorbonne University, Paris, France; c Department of Cardiac Surgery, Hôpitaux Universitaires de Strasbourg, Nouvel Hôpital Civil, Strasbourg, France

**Keywords:** Atrial septostomy, Cardiac assist device implantation, Left ventricular unloading, Left ventricular decompression, Peripheral venoarterial extracorporeal membrane oxygenation, Refractory cardiogenic shock

## Abstract

**Background:**

This study systematically reviewed the safety and efficacy of atrial septostomy as a left ventricular (LV) unloading intervention in paediatric and adult patients undergoing peripheral venoarterial extracorporeal membrane oxygenation (VA-ECMO).

**Methods:**

The PubMed, Cochrane, and Google Scholar online databases were searched, and studies describing patients who received VA-ECMO for refractory cardiogenic shock and underwent atrial septostomy for LV unloading were included. Laboratory experiments, animal studies, and patients who received ECMO with a method other than atrial septostomy for LV unloading were excluded.

**Results:**

From the 12 studies analysed, data were collected on 197 patients, including 97 (49 %) males and 75 (38 %) females (data unavailable for 25 patients) with ages ranging from 3.65 days to 70 years. VA-ECMO duration was 1.71 to 40 days (*P* < 0.001). Weaning from VA-ECMO with LV discharge was achieved successfully in 126 (64 %) patients, with 60 (30.5 %) in recovery (*P* = 0.006) and 66 (33.5 %) converted to a ventricular assistant device or transplantation. Additionally, 54 (27.4 %) patients experienced unsuccessful weaning. During atrial septostomy for LV unloading, 14 (7.1 %) patients experienced complications, whereas 180 (91.4 %) did not (*P* = 0.250). After LV unloading in patients receiving VA-ECMO, 60 (30.5 %) experienced early mortality (*P* = 0.286).

**Conclusion:**

VA-ECMO-assisted percutaneous atrial septostomy is a viable, safe, and successful alternative for LV unloading in both children and adults with refractory cardiogenic shock. However, further studies with larger sample sizes are required to comprehensively assess the morbidity and mortality associated with this approach.

## Graphical abstract

**Figure:**
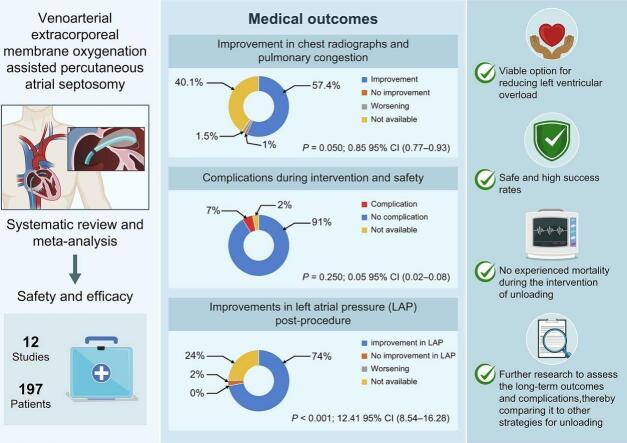

## Abbreviations

ASD: atrial septal defect
BAS: Balloon atrial septostomy
IRQ: interquartile range
LA: left atrial
LAP: left atrial pressure
LV: left ventricular
PBAS: percutaneous balloon atrial septostomy
TALVV: transapical LV vent
VA-ECMO: venoarterial extracorporeal membrane oxygenation

## 1 Introduction

Peripheral venoarterial extracorporeal membrane oxygenation (VA-ECMO) is used as cardiopulmonary and circulatory support in patients with refractory cardiogenic shock [1]. The use of VA-ECMO has increased substantially over time, with growing clinical indications in centres with experienced physicians that are proficient with and capable of performing ECMO [2,3]. VA-ECMO is a temporary resuscitation intervention with use limited to days or weeks until the patient achieves native heart recovery or receives a definitive long-term solution, such as cardiac assist device implantation or heart transplantation [4,5]. VA-ECMO provides urgent cardiopulmonary support for patients with refractory cardiogenic shock mainly by cannulation of the femoral vessels [6].

However, currently, mortality rates after support with VA-ECMO range from 28 to 42 % [4]. Moreover, VA-ECMO potentially negatively impacts the left ventricle (LV) [5] by retrograde flow from the aorta to the LV, increasing LV afterload and triggering LV dysfunction, which prevents the left side of the heart from performing typical tasks [7]. This pertains to the case of peripheral VA-ECMO, as open chest VA-ECMO with central cannulation would have minimal impact on the afterload [8].

The adverse effects of LV pressure overload include LV dilatation, increased left atrial (LA) pressure, and pulmonary oedema [9]. Additionally, during VA ECMO, the LV is exposed to distension by blood from different sources. These include possible aortic regurgitation, flow from Thebesian veins into the myocardium, recirculation of bronchial perfusion, and most importantly, any of the systemic venous blood that escapes aspiration by the ECMO venous cannula. These two features of retrograde flow and multiple sources of blood entry account for the complex hemodynamic challenges in mechanical assistance with VA ECMO [10].

In consequence, LV overload increases wall tension and myocardial oxygen consumption, thereby compromising ventricular recovery, particularly in the presence of ischaemia-induced myocardial impairment [9]. The aortic valve may remain closed during high VA-ECMO flow when extreme overload severely compromises LV contractility [11]. This causes blood stagnation, leading to thrombus formation [11]. Pulmonary oedema also occurs due to ventricular dilatation, which exacerbates LA overload, particularly in patients with chronic heart failure and severe valvular regurgitation [12]. These adverse events have been observed in animal models and validated in human studies [13].

Performing LV unloading during VA-ECMO satisfactorily reduces LA and LV congestion and ventricular dilatation, and resolves severe pulmonary oedema [14]. Additionally, early LA decompression has been reported to improve LV recovery [15]. However, the optimal method and target patient group most likely to benefit from venting interventions remain unclear [9].

Various approaches can achieve LV decompression; intra-aortic balloon pumping is a frequently utilised technique, although it has not been shown to improve survival rates [16]. Alternative methods include the use of axial flow pumps, such as the Abiomed Impella device [17] and TandemHeart system [18], as well as transaortic and percutaneous or surgical pulmonary artery or vein venting [19,20]. Lastly, minithoracotomy or percutaneous atrial septostomy can achieve LA decompression [21,22].

The LA unloading technique, usually performed using atrial septostomy, has been demonstrated in the paediatric population [15,21,[23], [24], [25], [26], [27]] and its use as LV unloading technique in adults is increasing [1,14,24,25,[27], [28], [29], [30]]. Atrial septostomy is a minimally invasive approach for preventing the adverse events of VA-ECMO and improving its duration and outcomes [1,14,24,25,[27], [28], [29], [30]]. Previous studies on LV unloading only examined the quantitative effects of this strategy on LV decompression [31]. Therefore, we conducted this systematic review and meta-analysis to evaluate the safety and efficacy of atrial septostomy as an LV unloading intervention in paediatric and adult patients undergoing VA-ECMO by improving LA pressure, pulmonary congestion, and chest radiographs. This review will help physicians working at centres that use VA-ECMO to choose the appropriate method to decompress the LV.

## 2 Materials and methods

### 2.1 Ethical statement

Ethical approval was not required

### 2.2 Search criteria

We systematically reviewed studies published between 1993 and 2022 using the PubMed, Cochrane, and Google Scholar online databases. The inclusion criteria were paediatric and adult patients who received VA-ECMO for refractory cardiogenic shock and underwent atrial septostomy for LV unloading. The exclusion criteria were animal studies, laboratory experiments, and patients who had received VA-ECMO with a modality of LV unloading other than atrial septostomy. The following terms were used: venoarterial extracorporeal membrane (venoarterial extracorporeal membrane oxygenation) or (extracorporeal cardiopulmonary resuscitation), left ventricular overload (left ventricular dilatation) or (left ventricular enlargement), left ventricular venting (left ventricular unloading) or (left ventricular decompression), atrial septostomy (limited atrial septostomy) or (balloon atrial septostomy), and percutaneous left ventricular or left atrial venting (unloading or decompression).

**Fig. 1:**
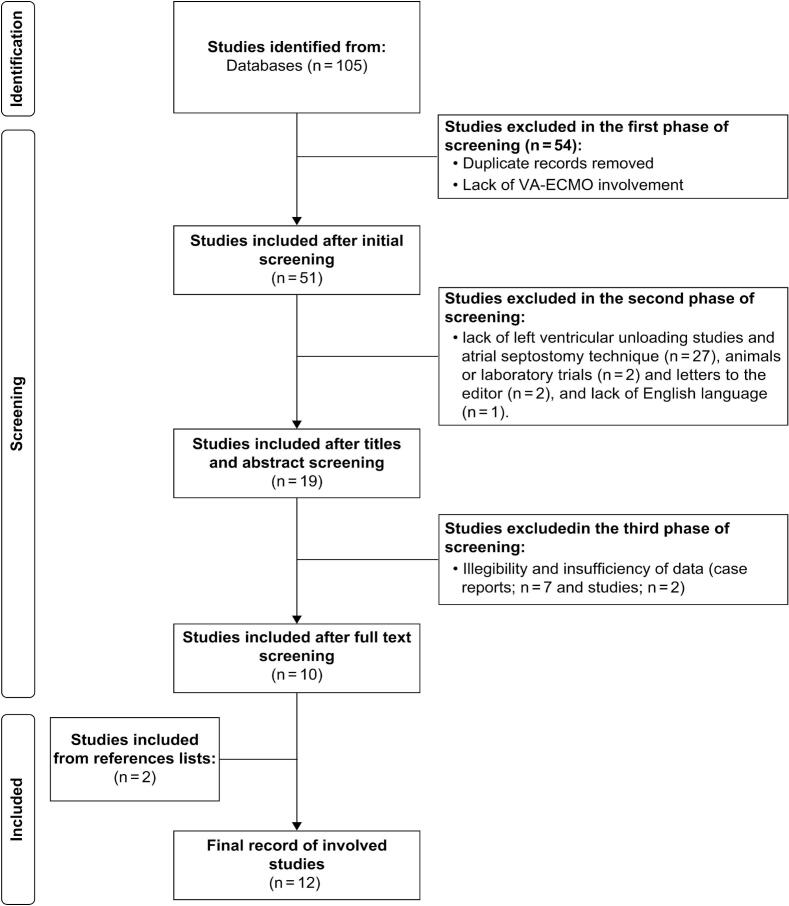
Flow chart of identified, screened, and included studies in the meta-analysis.

A total of 105 titles and abstracts were identified, which were screened in three stages (Fig. 1). At the initial screening of titles and abstracts, 54 articles were excluded because of duplication or lack of VA-ECMO involvement. In the second screening, 32 articles were excluded as they were animal or laboratory trials, letters to the editor, not written in English, not LV unloading studies, or employed a technique other than atrial septostomy for LV unloading. During the third screening, the full-text reports were reviewed to assess the eligibility and availability of sufficient data on atrial septostomies. Seven case reports were excluded because they were less comprehensive and lacked relevant data. Finally, two additional articles from the reference lists were included in the manual search. After completing the three-phase process, a final list of articles was created.

No publication date or publication status limits were imposed during the research process. Overall, we investigated the effects of LV unloading during VA-ECMO using the atrial septostomy method from 12 articles published between 1993 and 2022.

### 2.3 Statistics

In this single-arm meta-analysis, data were extracted from 12 articles, encompassing 197 study participants. The data presentation varied across studies; some reported means with standard deviations and others reported medians with interquartile ranges (IQRs), based on the metrics available in the original publications. Given the nature of our analysis, which focused on aggregating outcomes from single-arm studies without a direct comparator group, we employed a standardised approach for data synthesis. When medians were reported with the IQR, means and standard deviations were calculated using a validated formula to ensure consistency in our pooled analysis [32]. Statistical analyses were conducted using RevMan Version 5.4 and JAMOVI Version 2.4.2 software. Heterogeneity among effect estimates was assessed with Tau statistics and I^2^. For high heterogeneity, random effects models were employed during meta-analysis, while fixed effects models were used for low heterogeneity. Funnel plot asymmetry was evaluated through rank correlation and regression tests, along with Egger's test to investigate the possibility of publication bias. A *P*-value below 0.05 was considered statistically significant.

### 2.4 Assessment of risk of bias and quality of reporting

The risk of bias in the included studies is shown in **Supplementary Fig. S1**. Most studies exhibited a low level of risk of bias.

## 3 Results

### 3.1 VA-ECMO patient and procedure characteristics

Among the 12 studies selected for analysis (11 retrospective studies and 1 case report), data from 197 patients were collected. These patients varied in age from 3.65 days to 70 years. Of the total, 97 (49 %) were male, 75 (38 %) were female, and data on sex were unavailable for 25 (13 %) patients (**Supplementary Table S1**). The cohort consisted of 93 adult patients (47.2 % of total), 87 paediatric patients (44.1 %), and 17 patients whose age category was undetermined (8.6 %).

The timing of LV decompression initiation ranged from 0 to 12 days after the start of VA-ECMO (Table 1). VA-ECMO duration ranged from 1.71 to 40 days, with a mean of 12.1 ± 4.45 days (Table 2). The pooled mean duration of VA-ECMO (95 % confidence interval [CI]) was 11.95 days (8.86–15.03), utilising a random effects model with a restricted maximum likelihood estimator. Heterogeneity tests revealed that Tau, I^2^, and H^2^ were 4.081, 85.6 %, and 6.947, respectively (*P* < 0.001), as illustrated in the forest plot (**Supplementary Fig. S2**). Rank correlation (*P* = 0.109) and Egger's regression (*P* = 0.085) tests for funnel plot asymmetry indicated an absence of publication bias. VA-ECMO was performed for various indications. Weaning from VA-ECMO with LV discharge was successfully achieved in 126 (64 %) out of 180 patients, with 60 (30.5 %) patients recovering and 66 (33.50 %) patients being converted to a ventricular assistant device (VAD) or transplantation. Regrettably, 54 (27.4 %) patients failed to wean successfully. The early mortality rate for individuals who received VA-ECMO and underwent LV unloading was 60 (30.5 %) (Table 2).

**Table 1:** Description of atrial septostomy intervention as a method of LV unloading in VA-ECMO patients.

Study	Number	Age (years) median	Time of ECMO-atrial septostomy (days)	Indication for ECLS	Indication for left heart unloading	Type of balloon	Size of the balloon (mm)	Complication (%) 14 (7 %)	No. complications (%) 180 (91 %)
Koenig 1993 [21]	4	0.03 (0.01–5) ^1^	2.5 (0.5–3) ^1^	Acute myocarditis	LV and/or LA hypertension	Blade (1 patient) and Angioplasty balloon (3 patients)	N/A	0 (0)	4 (100)
Johnston 1999 [23]	1	10 (single case)	N/A	Myocarditis	Elevated LV end-diastolic pressure and/or pulmonary hypertension	Puncture transseptal with needle and angioplasty balloon	20	0 (0)	1 (100)
Seib 1999 [24]	10	3 (1–24) ^1^	N/A	Varied	LA / LV distension, pulmonary oedema/haemorrhage, or severe mitral regurgitation	Blade and Angioplasty balloon	[[12], [13], [14], [15]] ^4^ in pediatrics [[18], [19], [20]] ^4^ in adults	4 (40)	6 (60)
Kotani 2012 [15]	4	0.48 (0.03–10.8) ^1^	0.33 (0.17–0.5) ^1^	Varied	LA/LV dilatation and/or significant lung oedema	Angioplasty balloon	16.5 (13–18) ^1^	1 (25)	0 (0)
O'Byrne 2015 [26]	37	6 (0.01–17) ^1^	0 (0–2) ^1^	Varied	Pulmonary oedema and/or haemorrhage or elevated LA pressure	Angioplasty balloon	18 (14–18) ^1^	0 (0)	37 (100)
Eastaugh 2015 [25]	17	12.2 (0.02–18.9) ^1^	N/A	Varied	Pulmonary oedema/haemorrhage or left heart distension	Angioplasty balloon	12 (10–24) ^1^	0 (0)	17 (100)
Baruteau 2016 [27]	64	18 (0.3–72) ^1^	1.46 (0−12) ^1^	Varied	pulmonary oedema/haemorrhage and LV distension	Angioplasty balloon or Inoue balloon	28 (6–30) ^1^	6 (9)	58 (91)
Lin 2017 [14]	15	51 (22–65) ^1^	3 (1−11) ^1^	Varied	Refractory pulmonary oedema	Inoue balloon	24 or 26/27	0 (0)	15 (100)
Alhussein 2017 [28]	7	28 (21–50) ^1^	1 (0–2) ^1^	Varied	Pulmonary oedema	Angioplasty balloon+ LA cannula insertion (Percutaneous *trans*-Septal approach)	8 × 20 ^5^	0 (0)	7 (100)
Prasad 2018 [29]	9	46 (31–68.5) ^1^	N/A	Varied	Pulmonary edema and Upper body hypoxemia	Angioplasty balloon	(10–18 mm) ^4^	0 (0)	9 (100)
Hasde 2020 [30]	17	54.9 ± 16.0 ^3^	1.92 ± 0.15 ^3^	Varied	Pulmonary edema, increased pulmonary artery diastolic blood pressure or increased PCWP	Angioplasty balloon	N/A	3 (18)	14 (82)
Amancherla 2021 [1]	12	48 (IQR 15.5) ^1^	N/A	Varied	Left heart congestion	Limited angioplasty balloon	14.5 (12–18) ^1^	0 (0)	12 (100)

^⁎^ Values are expressed in ^1^ median, ^2^ mean, ^3^ mean ± SD, ^4^ range, ^5^ an 8-mm by 20-mm MUSTANG™ (Boston Scientific, Galway, Ireland) balloon has been used.

Abbreviations: BAS, balloon atrioseptostomy; LA, left atrium; LV, left ventricle; ECMO, extracorporeal membrane oxygenation; ECLS, extracorporeal life support; PCWP, pulmonary capillary wedge pressure; N/A, not available.

**Table 2:** Consequences following LV unloading with the atrial septostomy in VA-ECMO patients.

Study	No. of patients	Total running time ECMO (median) days	Total running time ECMO (mean) days	Weaning groups	Recovery	Bridge to definitive therapy	Weaning failure	Late mortality in successful weaning and unsuccessful weaning
Koenig 1993 [21]	4	7.5 (4–8) 1	6.75 ± 1.89 2	3	3	0	1	1
Johnston 1999 [23]	1	*N/A*	*N/A*	0	0	0	1	1
Seib 1999 [24]	10	9.17 (1.71–29.33) 1	11.89 ± 9.56 2	7	4	3	3	3
Kotani 2012 [15]	47	*N/A*	*N/A*	3	3	0	1	1
O'Byrne 2015 [26]	37	7 (2−123) 1	14.4 ± 19.13 2	30	21	9	7	7
Eastaugh 2015 [25]	17	*N/A*	*N/A*	0	0	0	0	0
Baruteau 2016 [27]	64	9 (4–24) 1	9.75 ± 4.28 2	48	17	31	16	23
Lin 2017 [14]	15	15 (5–40) 1	17.53 ± 10.11 2	9	3	6	6	7
Alhussein 2017 [28]	7	5 (2–9) 1	5.43 ± 2.44 2	5	1	4	2	2
Prasad 2018 [29]	9	14 (10−21) 1	14.65 ± 3.68 2	4	2	2	5	5
Hasde 2020 [30]	17	16 (11–23.5) 1	16.40 ± 3.48^2^	9	3	6	8	8
Amancherla 2021 [1]	12	*N/A*	*N/A*	8	3	5	4	5
Total (%)	197			126 (64.0)	60 (30.5)	66 (33.5)	54 (27.4)	63 (32.0)

Abbreviations: LV, left ventricular; VA-ECMO, venoarterial extracorporeal membrane oxygenation; N/A, outcome not available.

^⁎^ Values are expressed as

1 median

2 mean ± SD.

The use of atrial septostomy as a method of LV unloading in VA-ECMO patients is described in Table 1. This technique is commonly performed percutaneously, guided by echocardiography and fluoroscopy, in specialised medical centres with a high level of expertise [1,14,15,21,[23], [24], [25], [26], [27], [28], [29], [30]]. Atrial septostomy effectively relieves left-sided cardiac compression, reducing left atrial pressure (LAP) and improving pulmonary congestion. Additionally, this procedure has also been shown to enhance chest radiographs in both paediatric and adult patients on VA-ECMO support [1,14,15,21,[23], [24], [25], [26], [27], [28], [29], [30]].

### 3.2 Atrial septostomy for LV unloading in VA-ECMO patients improves chest radiographs and decreases signs of pulmonary congestion

Of the 197 patients included in this meta-analysis, 118 (59.9 %) had a follow-up chest radiograph after undergoing LV unloading by atrial septostomy, whereas 79 (40.1 %) did not. Following atrial septostomy, a total of 113 patients (57.4 %) demonstrated improvement in their chest radiographs, while 2 patients (1 %) showed no improvement, and 3 patients (1.5 %) experienced a worsening of their conditions (**Supplementary Table S2**). The deterioration was attributed to pneumonia and adult respiratory distress syndrome in one case [14] and unclear causes in two cases [1].

**Fig. 2:**
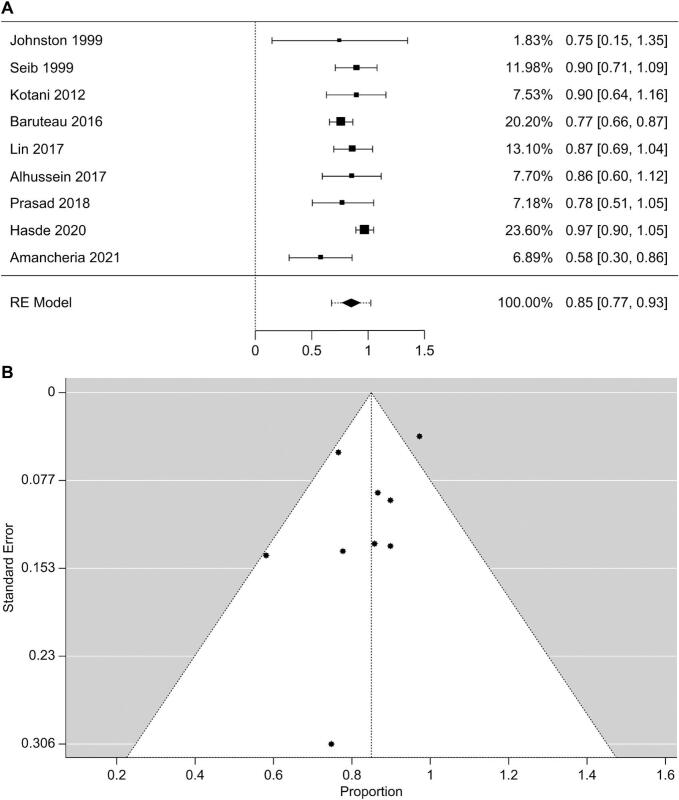
Improvements in chest radiographs. (A) Forest plot showing the intervention improved chest radiographs and (B) Funnel plot. Among the 12 studies screened, Koenig, et al. [21], O'Byrne, et al. [26] and Eastaugh, et al. [25] did not report any data regarding improvement in chest radiographs, and therefore were excluded from the analysis. The total sample size in the remaining 9 included studies was 139. The pooled improvement in chest radiograph (95 % CI) following the intervention was found to be 0.85 (0.77–0.93). A random effects model using a restricted maximum likelihood estimator was used. The heterogeneity tests showed Tau at 0.079, I2 at 45.67 %, and H2 at 1.841, with a *P* value of 0.050, as illustrated in the forest plot in Fig. 2A. Publication bias is absent according to rank correlation (*P* = 0.260) and regression (*P* = 0.266) tests for funnel plot asymmetry (Fig. 2B).

Out of 12 studies, Koenig (1993), O'Byrne (2015), and Eastaugh (2015) did not report any data regarding improvement in chest radiographs and were excluded from analysis [21,25,26]. The total sample size in the remaining nine included studies was 139 patients. The pooled improvement rate in chest radiographs (95 % CI) post-intervention was 0.85 (0.77–0.93) by a random effects model using a restricted maximum likelihood estimator. Heterogeneity tests showed that Tau, I^2^, and H^2^ were 0.079, 45.67 %, and 1.841, respectively, with a *P*-value of 0.050, as illustrated in the forest plot (Fig. 2A). The absence of publication bias was confirmed using rank correlation (*P* = 0.260) and regression (*P* = 0.266) tests for funnel plot asymmetry (Fig. 2B).

### 3.3 Complications and safety of atrial septostomy for LV unloading in VA-ECMO patients

Complications arose in 14 (7.11 %) patients who underwent atrial septostomy as a method of LV unloading, while 180 (91.37 %) of patients did not experience any complications [1,14,15,21,[23], [24], [25], [26], [27], [28], [29], [30]] (Table 1). There were no data available for 3 (1.52 %) of patients [15]. A summary of the main complications associated with atrial septostomy is presented in **Supplementary Table S3**.

All 12 studies were included in the meta-analysis with a sample size of 197 patients. The pooled complication rate (95 % CI) post-intervention was found to be 0.04 (0.01–0.06) by fixed effects model. The heterogeneity tests showed that Tau, I^2^, and H^2^ were 0, 19.71 %, and 1.246, respectively, with a *P*-value of 0.250, as illustrated in the forest plot (**Supplementary Fig. S3A**). There was an indication of publication bias, as per the rank correlation (*P* = 0.014) and regression (*P* = 0.005) tests for funnel plot asymmetry (**Supplementary Fig. S3B**).

### 3.4 Artificial atrial septal defect follow-up in VA-ECMO patients

Artificial atrial septal defect (ASD) follow-up results were available for 116 (58.9 %) of the patients, while 4 (2.03 %) were lost to follow-up, and 17 (8.6 %) had no data recorded (**Supplementary Table S4**). Percutaneous or surgical treatment of ASDs was performed in 12 (6.1 %) of the patients, with overt ASD noted during follow-up in 24 (12.2 %) patients without any intervention. Among the patients, 66 (33.5 %) received a VAD or heart transplantation, and 14 (7.1 %) had self-closed or no hemodynamically significant residual ASD. Early mortality was documented in 60 (30.5 %) patients during follow-up (**Supplementary Table S4**).

The total sample size in all the 12 included studies was 197. The pooled occurrence of persistent ASDs post-procedure (95 % CI) following intervention was found to be 0.08 (0.05–0.12) by fixed effects model. Heterogeneity tests showed that Tau, I^2^, and H^2^ were 0, 0 %, and 0.939, respectively, with a *P*-value of 0.501, as illustrated in the forest plot (**Supplementary Fig. S4A**). The absence of publication bias was confirmed through rank correlation (*P* = 0.113) and regression (*P* = 0.195) tests for funnel plot asymmetry (**Supplementary Fig. S4B**).

### 3.5 Atrial septostomy for LV unloading in VA-ECMO patients improves LAP post procedure

Out of a total of 197 patients, 146 (74 %) had improvement in LAP after undergoing LV unloading via atrial septostomy. Conversely, 4 (2 %) patients had no improvement, and 47 (24 %) patients had no records available of LAP measurements before or after the intervention (**Supplementary Table S5**). Notably, none of the patients experienced a worsening of LAP following the procedure. The mean LAP value before intervention was 29.83 ± 13.07 mmHg (95 % CI 21.05–38.60; *P* < 0.001) and it decreased to a mean of 16.16 ± 5.43 mmHg (95 % CI 12.51–19.81; P < 0.001) after the procedure (**Supplementary Table S5**).

Out of 12 studies, one study did not report LAP and was therefore excluded from this meta-analysis [25]. The total sample size in 11 included studies was 180. The pooled improvement in LAP (95 % CI) post procedure was found to be 12.41 mmHg (8.54–16.28) by a random effects model using a restricted maximum likelihood estimator. The heterogeneity tests showed that Tau, I^2^, and H^2^ were 6.145, 92.85 %, and 13.985, respectively, with a significant *P*-value (*P* < 0.001) as illustrated in the forest plot (**Supplementary Fig. S5A**). The presence of publication bias was confirmed by rank correlation (*P* = 0.026) and Egger's regression (*P* ≤0.001) tests for funnel plot asymmetry (**Supplementary Fig. S5B**).

### 3.6 Consequences following atrial septostomy for LV unloading in VA-ECMO patients

Successful weaning post-VA-ECMO with LV discharge was achieved in 126 (64 %) of 180 patients. Among them, 60 (30.5 %) fully recovered, while 66 (33.5 %) underwent conversion to VAD or transplantation. A total of 54 patients (27.4 %) experienced unsuccessful weaning (Table 2).

One study was excluded from the meta-analysis since it did not report any post-procedure mortalities [24]. The total sample size from the remaining 11 included studies was 180 patients. The pooled mortality rate (95 % CI) following intervention was found to be 0.35 (0.26–0.44) by a random effects model with a restricted maximum likelihood estimator. Heterogeneity tests showed that Tau, I^2^, and H^2^ were 0.074, 27.31 %, and 1.376, respectively, with a *P*-value of 0.286, as illustrated in the forest plot (**Supplementary Fig. S6A**). Publication bias was absent according to the rank correlation (*P* = 0.754) and regression (*P* = 0.205) tests for funnel plot asymmetry (**Supplementary Fig. S6B**).

### 3.7 Recovery of VA-ECMO patients post atrial septostomy procedure for LV unloading

One study did not report on any post-procedure recoveries [25] and was therefore excluded, resulting in a total sample size of 180 patients from the remaining 11 included studies. The pooled recovery rate (95 % CI) following intervention was found to be 0.33 (0.22–0.44) by a random effects model using a restricted maximum likelihood estimator. Heterogeneity tests showed that Tau, I^2^, and H^2^ were 0.138, 59.95 %, and 2.497, respectively, with a significant *P*-value of 0.006, as illustrated in the forest plot (**Supplementary Fig. S7A**). Publication bias was absent according to rank correlation (*P* = 0.159) and regression (*P* = 0.259) tests for funnel plot asymmetry (**Supplementary Fig. S7B**).

## 4 Discussion

In this study, we comprehensively reviewed the safety and efficacy of atrial septostomy as an LV unloading intervention in paediatric and adult patients undergoing VA-ECMO following improvement in LA pressure, pulmonary congestion, and chest radiographs. Our meta-analysis found that atrial septostomy for LV unloading in VA-ECMO patients improved chest radiographs (pooled improvement (95 % CI) of 0.85) and LAP (pooled improvement of 12.41 mmHg). Atrial septostomy had a pooled complication rate of 5 %, pooled occurrence of persistent ASDs of 8 %, pooled mortality rate of 35 %, and pooled recovery rate of 33 %. Our meta-analysis overall suggests that atrial septostomy is a viable LV unloading method.

The benefit of LV unloading is based on two main concepts: 1) a reduction in LA pressure and pulmonary oedema facilitates early lung recovery and shortens the duration of sedation and ventilation; and 2) improved LV wall tension improves subendocardial perfusion, minimises myocardial oxygen consumption, and subsequently aids in regaining LV function [14]. Both mechanisms have been demonstrated using atrial septostomy [14].

Another benefit of atrial septostomy is that it has been found to immediately improve both upper body oxygenation and pulmonary oedema in patients undergoing VA-ECMO [29]. This is relevant to differential hypoxemia, a common issue during VA-ECMO mechanical support, which results in the upper body being supplied with poorly oxygenated blood from the heart while the lower body receives well‑oxygenated extracorporeal membrane oxygenation blood [29]. The clinical significance of differential hypoxemia during VA-ECMO remains unclear although some putative effects have been suggested, e.g., adverse neurological events. Such events occur in approximately 10–16 % of patients receiving VA-ECMO, including brain death, seizures, infarction, and haemorrhage [33]. Only 26 % of patients with cerebral infarction survive while on VA-ECMO [29]. Although cerebral injury during VA-ECMO is influenced by multiple factors, both before and after cannulation, the frequent occurrence and severe consequences of cerebral injury warrant aggressive correction of underlying factors whenever possible.

A recent meta-analysis of observational studies using VA-ECMO showed LV unloading attenuated mortality [34]. However, the optimal treatment procedures remained unclear. Device- and patient-specific determinants must be considered when selecting a discharge strategy [34]. Additionally, it may be challenging to perform unloading via peripheral intervention using Impella or an IABP (Intra-Aortic Balloon Pump) in patients with significant peripheral arterial disease due to the enhanced risk of haemorrhage, vascular injury, and extremity ischaemia [1,34,35]. Compared to patients with IABPs, those with Impella devices face a higher risk of these complications [35,36]. Furthermore, Impella implants carry a significant risk of haemolysis and may increase the chance of infection [[37], [38], [39]].

According to Kim et al., weaning from VA-ECMO due to LV contractile recovery showed no significant difference between the LA-venting and control groups (20 [32.3 %] vs. 17 [27.4 %] patients). The median total VA-ECMO running time was longer in the venting group than in the non-venting group (237 vs. 71 h) and the intensive care unit and hospital stays were longer in the LA-venting group. However, there was no difference in in-hospital mortality between the two groups, indicating no distinct advantage of the LA-venting method [40].

Hasde et al. documented the effectiveness of the transapical LV vent (TALVV) method versus percutaneous balloon atrial septostomy (PBAS) and IABP strategies for minimising pulmonary arterial pressure (20.3 ± 4.3 mmHg; *P* < 0.001 in TALVV technique) [30]. The results showed that the LA diameter and central venous pressure reduction were highest with the TALVV technique (14.8 ± 3.2 mm and 7.4 ± 1.1 mmHg, respectively; P < 0.001). In addition, all methods significantly resolved pulmonary congestion, as assessed by chest radiography 48 h after LV decompression. Although TALVV was the most effective strategy for LV venting compared to PBAS and IABPs, it was associated with significant complications. No significant differences were observed in the duration of VA-ECMO support, intensive care unit stay, or hospitalisation between LV unloading strategies [30].

Patients withdrawn from VA-ECMO support without heart transplantation are at significant risk of sustained residual atrial communication, which may represent considerable right-sided volume overload or right-to-left shunt [26]. In light of this, it may be necessary to consider treating these residual lesions either during other surgeries in the operating room or independently by employing a percutaneous occlusion device. According to Amancherla et al., the limited balloon atrial septostomy (BAS) showed a significant difference in the size of the balloon used, which was smaller in this study than in other studies, resulting in a relatively more minor ASD that did not require closure or repair and would likely be hemodynamically well tolerated [1].

These studies demonstrating the adequacy of LV unloading by atrial septostomy are limited by several factors, such as their small sample size and the time-length to recovery for VA-ECMO or definitive therapies. Furthermore, they lacked extended aftercare for artificial ASD. Hence, there is a need for larger, well-designed studies that include data from long-term follow-up on the health of recovered native hearts and evaluate the efficacy of invasive and minimally invasive techniques for LV decompression in patients receiving VA-ECMO.

This systematic review and meta-analysis has some limitations. First, the patient sample size was relatively small, especially in some meta-analyses that could not include all studies, since some studies did not report certain data, e.g., radiographs, mortality. Second, we also found relatively high heterogeneity and/or publication bias in some analyses. Lastly, the heterogeneity of clinical conditions and patient characteristics among VA-ECMO recipients posed challenges for follow-up and comprehensive hemodynamic measurements, resulting in data gaps and incomplete follow-up in multiple studies. Moreover, a notable constraint of this review was its narrow focus on early mortality rates as the primary outcome measure. This limitation precluded the examination of other crucial endpoints, including long-term survival, quality of life, and cardiac function, due to insufficient data availability. Consequently, this restriction hindered a thorough assessment of the intervention's overall impact.

## 5 Conclusion

VA-ECMO-assisted percutaneous atrial septostomy is an effective alternative strategy for LV unloading in adults and children with refractory cardiogenic shock. It can be safely performed with a high degree of interventional success. This strategy needs to be further evaluated in studies with larger sample sizes to thoroughly assess morbidity and mortality. Additionally, a prospective, randomised controlled trial evaluating different LV decompression strategies would help compare indications, complications, and roles of each method in this diverse patient population.

## CRediT authorship contribution statement

**Mohammed AlGhamdi:** Writing – review & editing, Writing – original draft, Visualization, Validation, Software, Resources, Project administration, Methodology, Investigation, Formal analysis, Data curation, Conceptualization. **Gabriel Saiydoun:** Writing – review & editing, Writing – original draft, Visualization, Validation, Supervision, Methodology, Investigation, Formal analysis. **Guillaume Lebreton:** Writing – review & editing, Writing – original draft, Visualization, Validation, Supervision, Methodology, Investigation. **Jean-Philippe Mazzucotelli:** Writing – review & editing, Writing – original draft, Visualization, Validation, Supervision, Investigation, Formal analysis.

## Funding

The authors received no financial support for the research, authorship, and/or publication of this article.

## Declaration of competing interest

None declared.

## Data Availability

All data supporting this review are available online [1,14,15,21,[23], [24], [25], [26], [27], [28], [29], [30]].

## Appendix A Supplementary data

Supplementary material Image 1
