# Supplementary material for: Percutaneous atrial septostomy for left ventricular unloading in patients on peripheral venoarterial extracorporeal membrane oxygenation: A systematic review and meta-analysis
Source: Am Heart J Plus. 2025 Apr 9;54:100542. doi: 10.1016/j.ahjo.2025.100542 (PMC12019464; doi:10.1016/j.ahjo.2025.100542)
Supplement: Supplementary file 1 — Supplementary material [file mmc1.docx]

**Supplementary Materials**

**Supplementary Figures**

**
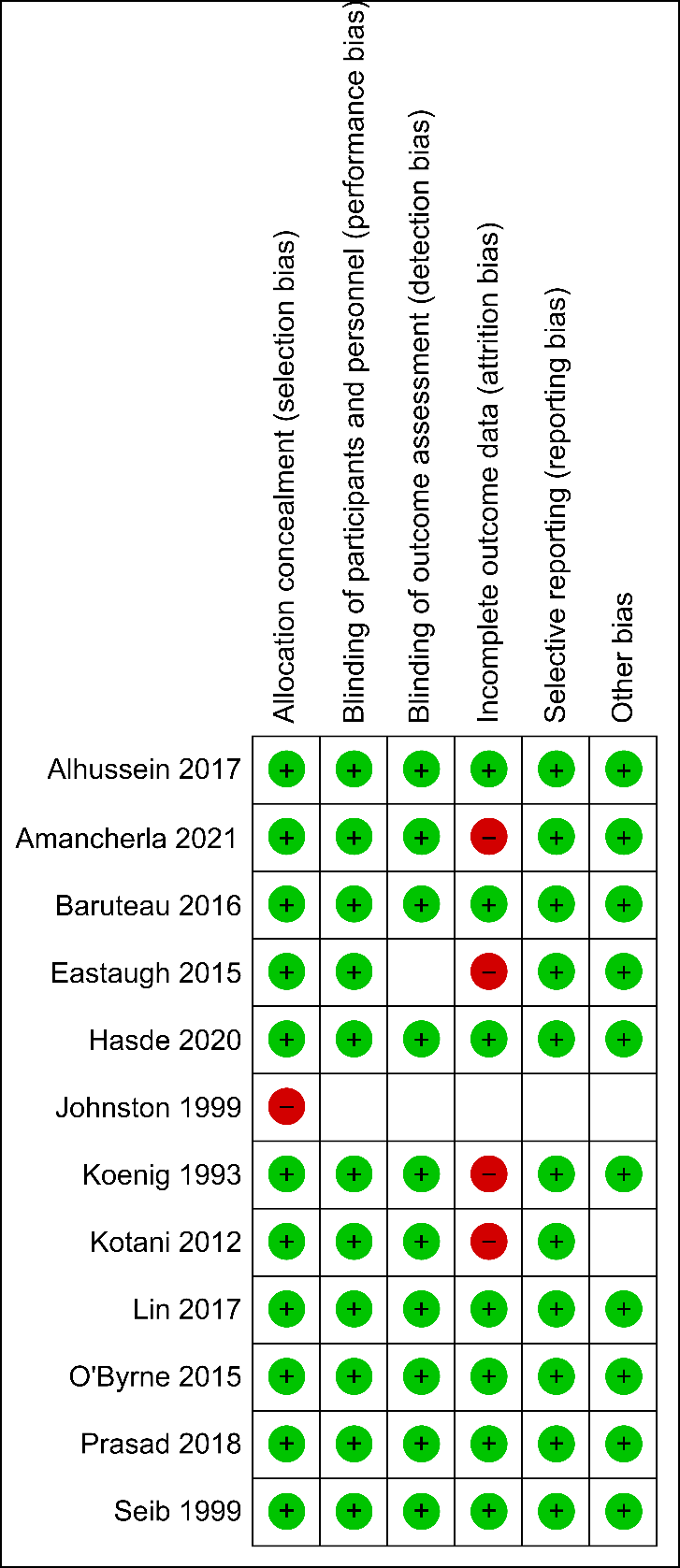
**

**Supplementary Figure S1. The risk of bias summary of studies included in the meta-analysis.**

**

**

**Supplementary Figure S2. (A): Forest plot showing the duration of VA ECMO. (B): Funnel Plot**

VA-ECMO duration: Out of 12 studies, 4 did not report VA-ECMO duration, and were, therefore, excluded from this meta-analysis leaving 8 included studies with a sample size of 163. The pooled mean VA-ECMO duration (95% CI) was 11.95 days (8.86–15.03). A random effects model using a restricted maximum likelihood estimator was used. Heterogeneity tests found Tau at 4.081, I2 at 85.61%, and H2 at 6.947, with significant P-value<0.001, as illustrated in the forest plot in Figure S2A. Publication bias was absent according to rank correlation (P=0.109) and Egger’s regression (P=0.085) tests for funnel plot asymmetry. The funnel plot is provided in Figure S2B.





**Supplementary Figure S3. (A): Forest plot showing the Complications during intervention. (B): Funnel Plot.** All 12 studies were included in this meta-analysis, with a sample size of 197. The pooled complication rate (95% CI) following the intervention was 0.04 (0.01–0.06). A fixed effects model was used. Heterogeneity tests found Tau at 0, I2 at 19.71%, and H2 at 1.246, with a P-value of 0.250, as illustrated in the forest plot in Figure S3A. Publication bias was present according to rank correlation (P=0.014) and regression (P=0.005) tests for funnel plot asymmetry. The funnel plot is provided in Figure S3B.





**Supplementary Figure S4. (A) Forest plot showing the presence of persistent atrial septal defects post procedure. (B) Funnel Plot.** Atrial septal defects post procedure: All 12 studies were included in this meta-analysis, with a sample size of 197. The pooled persistent atrial septal defects rate post procedure (95% CI) following the intervention was 0.08 (0.05–0.12). A fixed effects model was used. The heterogeneity tests found Tau at 0, I2 at 0%, and H2 at 0.939, with a P-value of 0.501, as illustrated in the forest plot in Figure S4A. Publication bias was absent according to rank correlation (P=0.113) and regression (P=0.195) tests for funnel plot asymmetry. The funnel plot is provided in Figure S4B.





**Supplementary Figure S5. (A) Forest plot showing the Improvements in left atrial pressure (LAP) post procedure. (B) Funnel Plot.** Out of 12 studies, 1 study did not report LAP, and was therefore excluded for this meta-analysis. The total sample size in the 11 included studies was 180. The pooled improvement in left atrial pressure (95% CI) post procedure was found to be 12.41 mmHg (8.54–16.28). A random effects model using a restricted maximum likelihood estimator was used. The heterogeneity tests showed Tau at 6.145, I^2^ at 92.85%, and H^2^ at 13.985, with a significant P value < 0.001, as illustrated in the forest plot in Figure S5A. Publication bias is present according to rank correlation (P = 0.026) and Egger’s regression (P = <0.001) tests for funnel plot asymmetry. The funnel plot is provided in Figure S5B.

**

**

**Supplementary Figure S6. (A) Forest plot showing post-procedure mortalities. (B) Funnel Plot.** One study was excluded from the meta-analysis since it did not report any post-procedure mortalities (26). The total sample size from the remaining 11 included studies was 180 patients. The pooled mortality rate (95% CI) following intervention was found to be 0.35 (0.26–0.44) by a random effects model with a restricted maximum likelihood estimator. Heterogeneity tests showed that Tau, I2, and H2 were 0.074, 27.31%, and 1.376, respectively, with a P-value of 0.286, as illustrated in the forest plot (Figure S6A). Publication bias was absent according to the rank correlation (P=0.754) and regression (P=0.205) tests for funnel plot asymmetry (Figure S6B).





**Supplementary Figure S7. A) Forest plot showing the recovery post procedure. (B) Funnel Plot.** The Eastaugh et al. (25) study did not report recovery post procedure and therefore excluded. The total sample size in the remaining 11 included studies was 180. The pooled recovery rate (95% CI) following the intervention was found to be 0.33 (0.22–0.44). A random effects model using a restricted maximum likelihood estimator was used. The heterogeneity tests showed Tau at 0.138, I^2^ at 59.95%, and H^2^ at 2.497, with a significant P value of 0.006, as illustrated in the forest plot in Figure S7A. Publication bias is absent according to rank correlation (P = 0.159), and regression (P = 0.259) tests for funnel plot asymmetry. The funnel plot is provided in Figure S7B.

**Supplementary Tables**

**Supplementary Table S1.** A demographics of patients involved in this study.

| **Study** | **Design** | **No. of patients** | **Population** | **Age (years)**  **median** | **Age (years)**  **mean** | **Sex** | | |  |
| --- | --- | --- | --- | --- | --- | --- | --- | --- | --- |
|  |  |  |  |  |  | **Male (%)** | **Female (%)** | **N/A (%)** | |
| Koenig 1993 (21) | Retrospective | **4** | Paediatric | 0.03 (0.01−5) ¹ | 1.27 ± 2.49 ^2^ | 0 (0) | 0 (0) | 4 (100) | |
| Johnston 1999 (23) | Case report | **1** | Paediatric | 10 (single case) | 10 | 1 (100) | 0 (0) | 0 (0) | |
| Seib 1999 (24) | Retrospective | **10** | Paediatric/ adult | 3 (1−24) ¹ | 6.86 ± 7.71 ^2^ | 4 (40) | 6 (60) | 0 (0) | |
| Kotani 2012 (15) | Retrospective | **4** | Paediatric | 0.48 (0.03−10.8) ¹ | 2.95 ± 5.23 ^2^ | 0 (0) | 0 (0) | 4 (100) | |
| O’Byrne 2015 (26) | Retrospective | **37** | Paediatric | 6 (0.01−17) ¹ | 6.53 ± 4 * ^2^ | 23 (62) | 14 (38) | 0 (0) | |
| Eastaugh 2015 (25) | Retrospective | **17** | Paediatric/ adult | 12.2 (0.02−18.9) ¹ | 11.31 ± 5.26 * ^2^ | 0 (0) | 0 (0) | 17 (100) | |
| Baruteau 2016 (27) | Retrospective | **64** | Paediatric/ adult | 18 (0.3 – 72) ¹ | 20.73 ± 15.34 * ^2^ | 33 (52) | 31 (48) | 0 (0) | |
| Lin 2017 (14) | Retrospective | **15** | Adult | 51 (22−65) ¹ | 48.27 ± 11.87 ^2^ | 9 (60) | 6 (40) | 0 (0) | |
| Alhussein 2017 (28) | Retrospective | **7** | Adult | 28 (21−50) ¹ | 33.14 ± 11.91 ^2^ | 4 (57) | 3 (43) | 0 (0) | |
| Prasad 2018 (29) | Retrospective | **9** | Adult | 46 (31– 68.5) ¹ | 47.63 ± 12.54 * ^2^ | 4 (44) | 5 (56) | 0 (0) | |
| Hasde 2020 (30) | Retrospective case-control study | **17** | Adult | 54.9 ± 16.0 ^2^ | 54.9 ± 16.0 ^2^ | 10 (59) | 7 (41) | 0 (0) | |
| Amancherla 2021 (1) | Retrospective | **12** | Adult | 48 (IQR 15.5) ^1^ | 48 (IQR 15.5) ^1^ | 9 (75) | 3 (25) | 0 (0) | |
| **Total** | **-** | **197** | **-** |  | **-** | **97 (49)** | **75 (38)** | **25 (13)** | |

* The values are expressed as (1) Median, (2) Mean ± SD.

*Median (25th to 75th interquartile range), it was converted to mean ± standard deviation using the website: https://www.math.hkbu.edu.hk/~tongt/papers/median2mean.html

| **Study** | **No. of patients** | **Improvement (%)** | **No Improvement (%)** | **Worsening (%)** | **Not available (%)** |
| --- | --- | --- | --- | --- | --- |
| Koenig 1993 (21) | **4** | 0 (0) | 0 (0) | 0 (0) | 4 (100) |
| Johnston 1999 (23) | **1** | 1 (100) | 0 (0) | 0 (0) | 0 (0) |
| Seib 1999 (24) | **10** | 9 (90) | 1 (10) | 0 (0) | 0 (0) |
| Kotani 2012 (15) | **4** | 4 (100) | 0 (0) | 0 (0) | 0 (0) |
| O’Byrne 2015 (26) | **37** | 0 (0) | 0 (0) | 0 (0) | 37 (100) |
| Eastaugh 2015 (25) | **17** | 0 (0) | 0 (0) | 0 (0) | 17 (100) |
| Baruteau 2016 (27) | **64** | 49 (76.6) | 0 (0) | 0 (0) | 15 (23.4) |
| Lin 2017 (14) | **15** | 13 (86.7) | 1 (6.67) | 1 (6.67) | 0 (0) |
| Alhussein 2017 (28) | **7** | 6 (85.7) | 0 (0) | 0 (0) | 1 (14.3) |
| Prasad 2018 (29) | **9** | 7 (77.8) | 0 (0) | 0 (0) | 2 (22.2) |
| Hasde 2020 (30) | **17** | 17 (100) | 0 (0) | 0 (0) | 0 (0) |
| Amancherla 2021 (1) | **12** | 7 (58.3) | 0 (0) | 2 (16.7) | 3 (25) |
| **Total** | **197** | **113 (57.4)** | **2 (1)** | **3 (1.5)** | **79 (40,1)** |

**Supplementary Table S2.** Chest radiography improvement after LV unloading using septostomy intervention.

**Supplementary Table S3.** An overview of the pertinent atrial septostomy intervention complications is provided below.

| **The relevant complications during the intervention of atrial septostomy:** | **No. of complications (%) *** |
| --- | --- |
| Post-intervention hypotension resolved by volume infusion | 2 (13.3) |
| Left atrial needle perforation without haemodynamic compromise | 4 (26.7) |
| Cardiac arrythmia | 5 (33.3) |
| Transient complete heart block | 1 (6.7) |
| Vascular complication | 2 (13.3) |
| Pericardial tamponade | 1 (6.7) |
| **Total** | **15** |

* The percentage values represent the proportion of each complication relative to the total number of complications.

**Supplementary Table S4**. Number of VA-ECMO patients who were monitored following the intervention and management of atrial septal defect (ASD) post LV unloading using atrial septostomy.

| Study | No. of patients | Follow up for living patients (%) | No follow up (lost follow up) (%) | Treated (%) | Patent ASD (%) | Transplanted or VAD (%) | Closed by its self or no hemodynamically significant residual ASD (%) | Early mortality (%) | N/A (%) |
| --- | --- | --- | --- | --- | --- | --- | --- | --- | --- |
| Koenig 1993 (21) | 4 | 3 (75) | 0 (0) | 0 (0) | 2 (50) | 0 (0) | 1 (25) | 1 (25) | 0 (0) |
| Johnston 1999 (23) | 1 | 0 (0) | 0 (0) | 0 (0) | 0 (0) | 0 (0) | 0 (0) | 1 (100) | 0 (0) |
| Seib 1999 (24) | 10 | 7 (70) | 0 (0) | 0 (0) | 1 (10) | 3 (30) | 3 (30) | 3 (30) | 0 (0) |
| Kotani 2012 (15) | 4 | 3 (75) | 0 (0) | 0 (0) | 0 (0) | 0 (0) | 3 (75) | 1 (25) | 0 (0) |
| O’Byrne 2015 (26) | 37 | 30 (81.1) | 0 (0) | 7 (18.9) | 9 (24.3) | 9 (24.3) | 5 (13.5) | 7 (19.0) | 0 (0) |
| Eastaugh 2015 (25) | 17 | 2 (11.8) | 1 (5.9) | 1 (5.9) | 1 (5.9) | 0 (0) | 0 (0) | 0 (0) | 14 (82,4) |
| Baruteau 2016 (27) | 64 | 42 (65.6) | 0 (0) | 2 (3.1) | 9 (14.1) | 31 (48.4) | 0 (0) | 22 (34.4) | 0 (0) |
| Lin 2017 (14) | 15 | 9 (60) | 0 (0) | 1 (6.7) | 2 (13.3) | 6 (40) | 0 (0) | 6 (40) | 0 (0) |
| Alhussein 2017 (28) | 7 | 4 (57.1) | 1 (14.3) | 0 (0) | 0 (0) | 4 (57.1) | 0 (0) | 2 (28.8) | 0 (0) |
| Prasad 2018 (29) | 9 | 3 (33.3) | 1 (11.1) | 0 (0) | 0 (0) | 2 (22.2) | 1 (11.1) | 5 (55.6) | 0 (0) |
| Hasde 2020 (30) | 17 | 6 (35.3) | 0 (0) | 0 (0) | 0 (0) | 6 (35.3) | 0 (0) | 8 (47.1) | 3 (17.7) |
| Amancherla 2021 (1) | 12 | 7 (58.3) | 1 (8.3) | 1 (8.3) | 0 (0) | 5 (41.7) | 1 (8.3) | 4 (33.3) | 0 (0) |
| Total | 197 | 116 (58.9) | 4 (2.0) | 12 (6.1) | 24 (12.2) | 66 (33.5) | 14 (7.1) | 60 (30.5) | 17 (8.6) |

**Abbreviations:** LV, left ventricular; VA-EMCO, venoarterial extracorporeal membrane oxygenation; VAD, ventricular assistant device; N/A, outcome not available.

**Supplementary Table S5.** Description of improvement in left atrial pressure after LV unloading using atrial septostomy.

| **Study** | **No. of patients** | **Improving in LAP (%)** | **No. improving in LAP (%)** | **Worsening (%)** | **No. available data (%)** | **Mean LAP (pre-procedure) mmHg** | **SD LAP (pre-procedure) mmHg** | **Mean LAP (Post-procedure) mmHg** | **SD LAP (Post-procedure) mmHg** |
| --- | --- | --- | --- | --- | --- | --- | --- | --- | --- |
| Koenig 1993 (21) | 4 | 1 (25) | 0 (0) | 0 (0) | 3 (75) | 20 | N/A | 12 | N/A |
| Johnston 1999 (23) | 1 | 1 (100) | 0 (0) | 0 (0) | 0 (0) | 65 | N/A | 28 | N/A |
| Seib 1999 (24) | 10 | 9 (90) | 0 (0) | 0 (0) | 1 (10) | 31.6 | 13.1 | 16.4 | 4.4 |
| Kotani 2012 (15) | 4 | 3 (75) | 1 (25) | 0 (0) | 0 (0) | 20.25 | 5.19 | 13.75 | 4.72 |
| O’Byrne 2015 (26) | 37 | 31 (84) | 2 (5) | 0 (0) | 4 (11) | 18.11 | 2.59 | 12.42 | 2.82 |
| Eastaugh 2015 (25) | 17 | 0 (0) | 0 (0) | 0 (0) | 17 (100) | N/A | N/A | N/A | N/A |
| Baruteau 2016 (27) | 64 | 64 (100) | 0 (0) | 0 (0) | 0 (0) | 24,2 | 6.9 | 7,8 | 2.6 |
| Lin 2017 (14) | 15 | 3 (20) | 0 (0) | 0 (0) | 12 (80) | 29 | N/A | 13 | N/A |
| Alhussein 2017 (28) | 7 | 1 (14) | 0 (0) | 0 (0) | 6 (86) | 35.5 | 4.95 | 18 | 0 |
| Prasad 2018 (29) | 9 | 9 (100) | 0 (0) | 0 (0) | 0 (0) | 33.31 | 4.69 | 20.78 | 5.02 |
| Hasde 2020 (30) | 17 | 17 (100) | 0 (0) | 0 (0) | 0 (0) | 29.3 | 2.5 | 19.7 | 2.5 |
| Amancherla 2021 (1) | 12 | 7 (58) | 1 (9) | 0 (0) | 4 (33) | 21.8 | 10.1 | 15.9 | 7.7 |
| **Total** | **197** | **146 (74)** | **4 (2)** | **0 (0)** | **47 (24)** | **-** | **-** | **-** | **-** |

**Abbreviations:** LAP: left atrial pressure, SD: standard deviation.
